# Knockout of c-Cbl/Cbl-b slows c-Met trafficking resulting in enhanced signaling in corneal epithelial cells

**DOI:** 10.1016/j.jbc.2023.105233

**Published:** 2023-09-09

**Authors:** Kate Tarvestad-Laise, Brian P. Ceresa

**Affiliations:** Department of Pharmacology and Toxicology (KTL, BPC) and Department of Ophthalmology and Vision Sciences (BPC), University of Louisville, Louisville, Kentucky, USA

**Keywords:** cornea, Cbl-b, receptor tyrosine kinase, receptor desensitization, wound healing, c-Cbl, c-Met

## Abstract

In many cell types, the E3 ubiquitin ligases c-Cbl and Cbl-b induce ligand-dependent ubiquitylation of the hepatocyte growth factor (HGF)–stimulated c-Met receptor and target it for lysosomal degradation. This study determines whether c-Cbl/Cbl-b are negative regulators of c-Met in the corneal epithelium (CE) and if their inhibition can augment c-Met–mediated CE homeostasis. Immortalized human corneal epithelial cells were transfected with Cas9 only (Cas9, control cells) or with Cas9 and c-Cbl/Cbl-b guide RNAs to knockout each gene singularly (-c-Cbl or -Cbl-b cells) or both genes (double KO [DKO] cells) and monitored for their responses to HGF. Cells were assessed for ligand-dependent c-Met ubiquitylation *via* immunoprecipitation, magnitude, and duration of c-Met receptor signaling *via* immunoblot and receptor trafficking by immunofluorescence. Single KO cells displayed a decrease in receptor ubiquitylation and an increase in phosphorylation compared to control. DKO cells had no detectable ubiquitylation, had delayed receptor trafficking, and a 2.3-fold increase in c-Met phosphorylation. Based on the observed changes in receptor trafficking and signaling, we examined HGF-dependent *in vitro* wound healing *via* live-cell time-lapse microscopy in control and DKO cells. HGF-treated DKO cells healed at approximately twice the rate of untreated cells. From these data, we have generated a model in which c-Cbl/Cbl-b mediate the ubiquitylation of c-Met, which targets the receptor through the endocytic pathway toward lysosomal degradation. In the absence of ubiquitylation, the stimulated receptor stays phosphorylated longer and enhances *in vitro* wound healing. We propose that c-Cbl and Cbl-b are promising pharmacologic targets for enhancing c-Met–mediated CE re-epithelialization.

The cornea is the most anterior portion of the eye. It is made up of three cellular layers (posterior to anterior: endothelium, stroma, and epithelium) divided by two interfaces (Descemet’s membrane and Bowman’s layer (1)). Injury to the most external layer, the epithelium, can occur in a multitude of ways, including physical insult, burns, or as a consequence of certain chemotherapeutics (*i.e.*, amivantamab, crizotinib) and diseases like diabetes (2, 3, 4, 5, 6, 7, 8, 9). Superficial wounds in healthy individuals can re-epithelialize in 48 to 72 h, although full healing can take several months to years (10). However, some individuals experience persistent recurrent wounds to the cornea that can lead to complications, such as corneal fibrosis, opacification, and blindness, if not healed properly or timely (11, 12). Corneal blindness is the fourth leading cause of vision loss worldwide, impacting millions of people of all ages around the globe. Its etiology has a wide range, and, unlike diseases such as glaucoma and macular degeneration, corneal blindness is often avoidable with the proper health care (13). Despite the widespread prevalence and impact of corneal injury and blindness, there are few Food and Drug Administration–approved drugs that aid in healing and promoting homeostasis of the cornea or corneal surface.

The development of new ocular therapies requires a more complete understanding of the mechanisms that govern corneal epithelial homeostasis. A fully intact corneal epithelium is required for proper vision and alleviating pain. Growth factor receptors have a critical role in maintaining the 5 to 7 epithelial cell layers. This is supported by the observation that many patients taking receptor tyrosine kinase (RTK) inhibitors as anticancer therapies experience corneal perturbations (2, 3, 4, 5, 6, 7, 8, 9). Under homeostatic conditions, tyrosine kinase receptors promote cellular proliferation, migration, and differentiation, and when receptor activity is disrupted, corneal homeostasis is imbalanced. c-Met, also known as hepatocyte growth factor (HGF) receptor, is part of the RTK family and responds to HGF. HGF is expressed in basal tear fluid (∼6.5 pM; (14)) at concentrations lower than its reported *K*_*d*_ for c-Met (17–300 pM; (15, 16, 17, 18)). However, upon corneal epithelial debridement in mice, Wilson *et al.* (19) found that HGF mRNA expression in the lacrimal gland (where aqueous tears are produced) increased sevenfold 24 h postinjury. In addition, c-Met mRNA expression increased 7.6-fold from the basal levels. These taken together suggest an already-present physiological role for the HGF–c-Met signaling axis in corneal wound healing.

HGF-mediated c-Met activity in the corneal epithelium has shown promise *in vivo* (20, 21) as a restorative agent and has been recapitulated in both *ex vivo* (22, 23) and *in vitro* (24, 25) models. HGF promotes corneal epithelial proliferation while also decreasing inflammatory mediators *in vivo* (20) and decreases opacification of the cornea by inhibiting expression of transforming growth factor-β—a mediator of corneal fibrosis—in the stromal layer (26, 27, 28, 29, 30, 31, 32, 33). In addition, HGF aids in the *in vitro* and *in vivo* growth of nerves, possibly opening doors to more comprehensive corneal healing process if used clinically (34, 35, 36). Aside from the cornea, HGF has shown to aid in repair of the liver (37), kidney (38), lung (39), gastrointestinal mucosa (40), and skin (41).

All this considered, HGF presents as a viable therapeutic that can aid in multiple facets of corneal epithelial healing. However, addition of exogenous HGF to the cornea has not always shown promise. Previous *in vivo* studies investigating c-Met signaling that used supraphysiologic concentrations of HGF (0.1 mg/ml), three times per day for 1 week following six doses the day of the surgery, provided no therapeutic benefit with regard to epithelial wound closure rate or decreased opacification, failing to demonstrate the therapeutic benefit of the growth factor (42). We hypothesize that this is due to attenuated signaling through receptor desensitization and degradation.

Desensitization is a negative regulator of receptor signaling in response to sustained ligand stimulation. One mechanism of desensitization is ligand-mediated receptor endocytosis (18). The progression of the ligand–receptor complex from the plasma membrane through the endocytic pathway is controlled by various regulatory proteins (43). Inhibition of these regulatory proteins is a novel strategy to sustain receptor signaling. Ubiquitin post-translational modifications have many functions in the cell. For RTKs, it confers receptor internalization from the cell surface and/or targeting proteins for degradation in the lysosome to terminate receptor signaling (44, 45, 46). Previous studies have demonstrated that c-Cbl activity is required for the normal kinetics of c-Met degradation following HGF activation (47, 48, 49). E3 ligases are an attractive therapeutic target because of their substrate specificity and the requirement of ligand-mediated receptor activation for their mobilization (50, 51).

In this study, we test the hypothesis that inhibition of two structurally similar E3 ubiquitin (Ub) ligases (*i.e.*, c-Cbl and Cbl-b) in corneal epithelial cells will enhance HGF-driven c-Met responses. Using inhibition of c-Cbl and Cbl-b expression by CRISPR–Cas9 knockout, we observed a decrease in the amount of detectable c-Met following Ub immunoprecipitation, slowed trafficking of the HGF–c-Met complex, and promoted c-Met-mediated *in vitro* wound healing. Thus, inhibition of c-Met ubiquitylation by blocking c-Cbl and Cbl-b activity is a promising new target to enhance corneal epithelial homeostasis.

## Results

**Figure 1:**
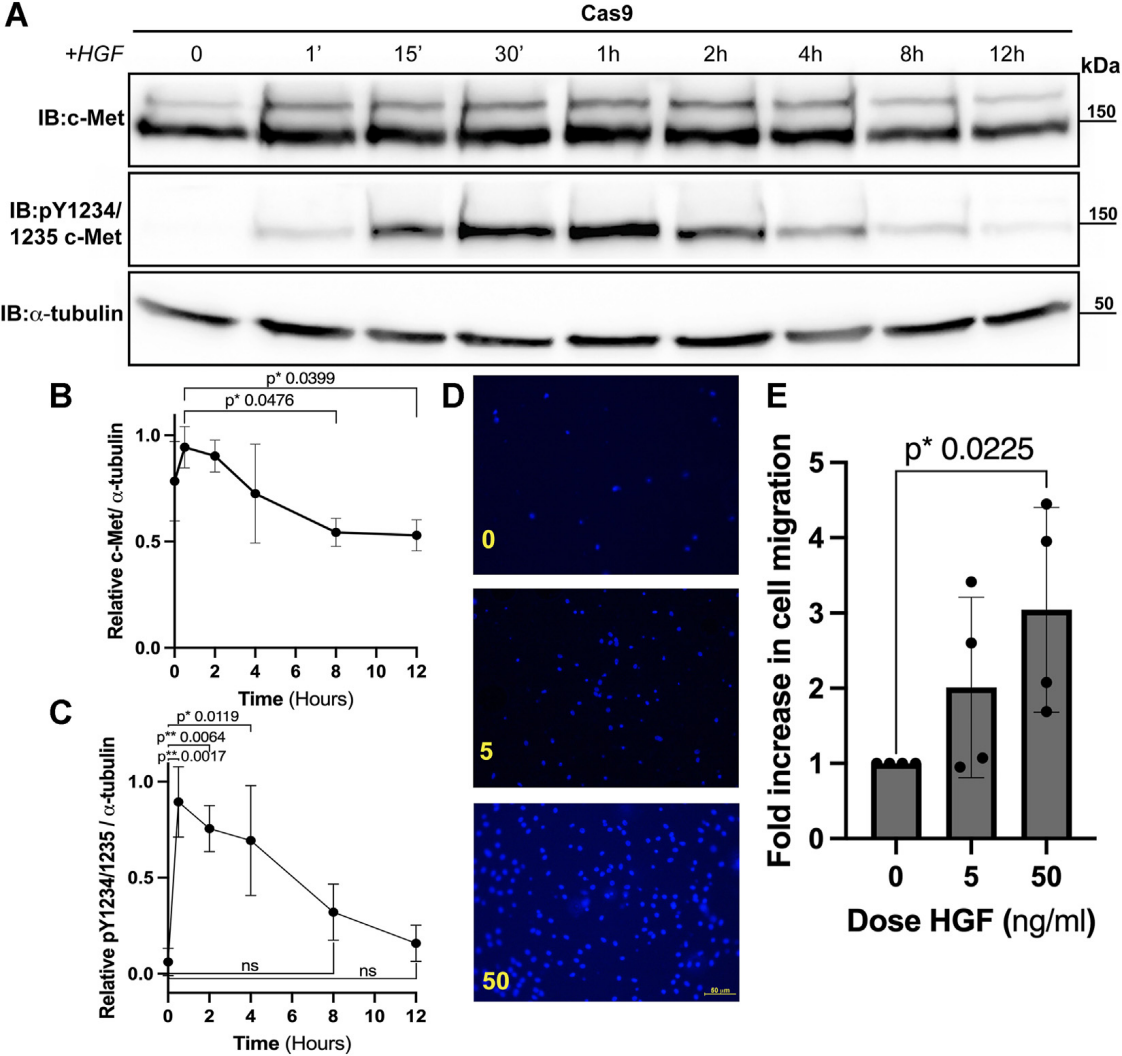
**Treatment of human corneal epithelial cells with HGF results in c-Met receptor phosphorylation, receptor degradation, and cell migration.***A*, Cas9 cells were treated with HGF (50 ng/ml) and harvested at the indicated time points (0–12 h). Lysates (20 μg) were resolved by 10% SDS-PAGE, transferred to nitrocellulose, and membranes were immunoblotted for total c-Met, phosphorylated c-Met (pY1234/1235), and α-tubulin as a loading control. Shown is a representative blot from an experiment performed three times. *B* and *C*, three independent experiments were subject to densitometry quantification using ImageJ. Graphed are the average ± SD. *B*, c-Met expression normalized to α-tubulin expression (N = 3). *C*, phospho-c-Met expression normalized to α-tubulin expression (N = 3). Data were analyzed *via* two-way ANOVA. *D*, Cas9 cells were seeded in transwell inserts and placed in wells containing increasing doses of HGF for 16 h. Membranes were washed and fixed in methanol. Cells on the upper membrane were removed with a cotton swab prior to mounting in DAPI-containing medium. Ten fields of view (20× magnification) per membrane were randomly selected, and cells were counted. Representative images are shown (N = 4). Scale bar represents 50 μm. *E*, cell counts were normalized to untreated conditions. Graphed are average ± SD and individual data points. Data were analyzed *via* two-way ANOVA. DAPI, 4′,6-diamidino-2-phenylindole; HGF, hepatocyte growth factor.

**Figure 2:**
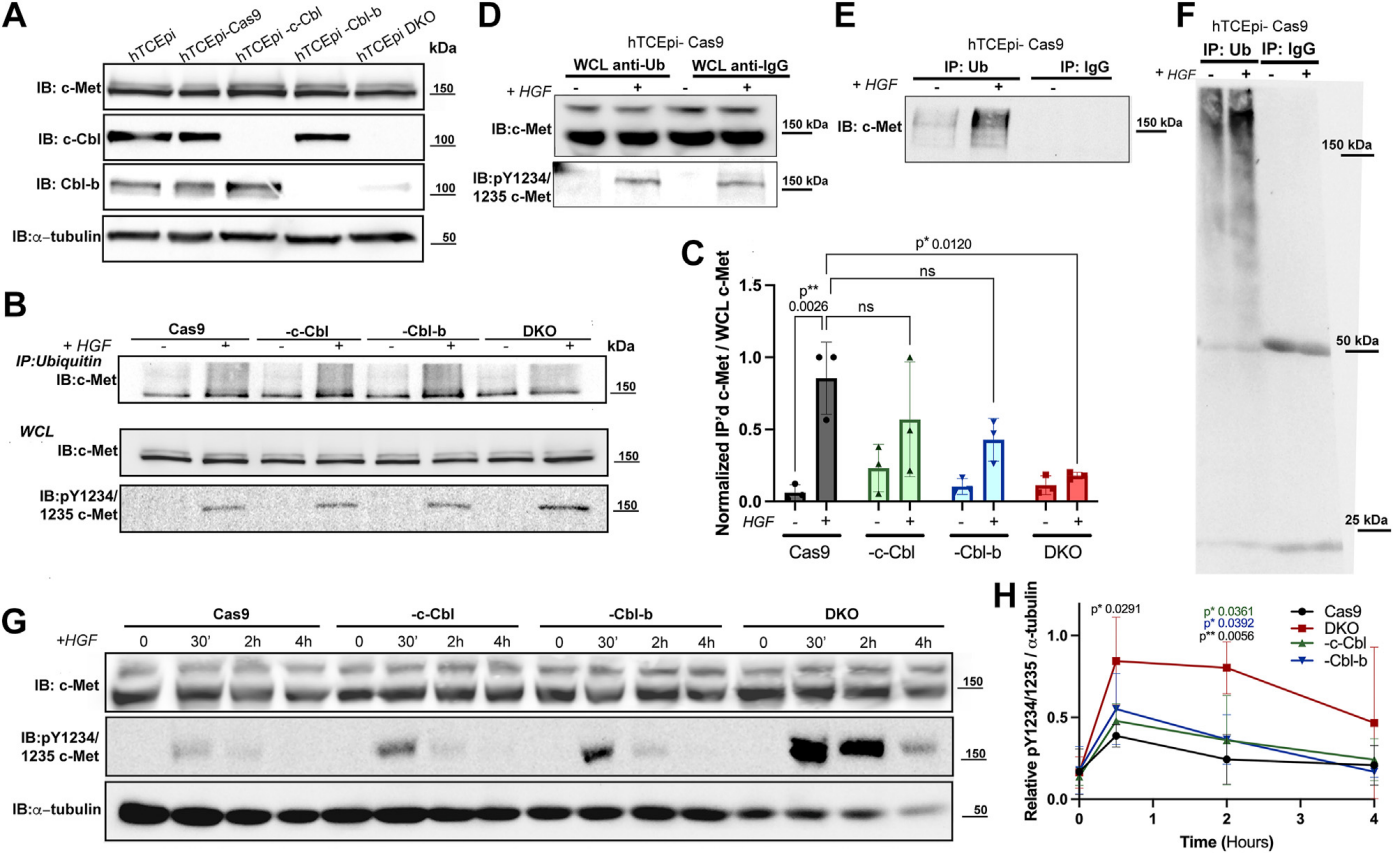
**Only double knockout of c-Cbl and Cbl-b significantly alters c-Met phosphorylation and detectable ubiquitylated c-Met.***A*, lysates were prepared from hTCEpi, hTCEpi Cas9, hTCEpi -c-Cbl, hTCEpi -Cbl-b, and hTCEpi -c-Cbl/-Cbl-b (DKO) cells. Lysates (20 μg) were resolved by SDS-PAGE, transferred to nitrocellulose, and immunoblotted using antibodies against total c-Met, c-Cbl, Cbl-b, and α-tubulin as a loading control. Shown are representative blots from an experiment repeated three times. *B*, lysates (20 μg) were prepared from hTCEpi Cas9, -c-Cbl, -Cbl-b, and DKO cells that were treated without (−) or with (+) HGF (50 ng/ml) for 30 min. Ubiquitylated proteins were immunoprecipitated using an antiubiquitin antibody. Immunoprecipitates (*upper panels*) were resolved by SDS-PAGE, transferred to nitrocellulose, and immunoblotted using antibodies against total c-Met. Whole cell lysates (WCLs) (*lower panel*) were probed for total c-Met and phosphorylated c-Met to ensure c-Met activation with HGF treatment. Shown are representative blots from an experiment repeated three times. *C*, immunoprecipitated c-Met densitometry was normalized to WCL c-Met densitometry. Graphed are average ± SD and individual data points. Data were analyzed *via* two-way ANOVA. *D*–*F*, Cas9 cells were pretreated with MG-132 and treated with or without HGF as indicated in methods. A portion of the WCL was kept for running separately. Lysates were either incubated with FK2 antiubiquitin (catalog no.: ST1200; MilliporeSigma) antibody or mouse immunoglobulin G (catalog no.: 5415; Cell Signaling Tech). Immunoprecipitated proteins were isolated using A/G Agarose beads as described in Experimental procedures section. Portions of the WCL (*D*) and immunoprecipitates (*E* and *F*) were resolved through SDS-PAGE and immunoblotted for ubiquitin (catalog no.: 8017; Santa Cruz), c-Met, and phosphorylated c-Met pY1234/1235 to ensure receptor activation. Our data indicate a ubiquitin-specific pulldown and are consistent with Schwertman *et al.* (58). *G*, hTCEpi Cas9, -c-Cbl, -Cbl-b, and DKO cells were treated with HGF (50 ng/ml) and harvested at the indicated time points (0–4 h). Lysates were resolved, and membranes were immunoblotted for total c-Met, phosphorylated c-Met (pY1234/1235), c-Cbl, Cbl-b, and α-tubulin. Shown are representative blots from an experiment repeated three times. *H*, phosphorylated c-Met immunoreactive bands in (*D*) were quantified and normalized to α-tubulin. Graphed are average ± SD and individual data points. Data were analyzed *via* a two-way ANOVA. *p* Values on (*E*) indicate statistical significance between Cas9 (*black*), -c-Cbl (*blue*), or -Cbl-b (*green*) and DKO cells. DKO, double KO; HGF, hepatocyte growth factor; HTCEpi, human telomerase–immortalized corneal epithelial cell.

To assess the role of the HGF–c-Met signaling axis, we first validated the relative expression and intact signaling by the receptor in human telomerase–immortalized corneal epithelial (hTCEpi (52)) cells. c-Met expression has been well documented in other corneal epithelial cell lines and *in vivo* (19, 24, 53, 54). All control cells in this study are hTCEpi cells only expressing the Cas9 and blasticidin-resistance gene without guide or transactivating CRISPR RNA (tracrRNA), referred to as Cas9 cells. Expression of Cas9 did not cause any discernable changes in cell morphology or growth rate levels compared with the parental hTCEpi cells (Fig. S1). Receptor signaling in Cas9 cells was intact as evidenced by the phosphorylation of the receptor in the presence of HGF (Fig. 1, *A* and *C*) as well as the dose-dependent increase in cell migration with HGF treatment (Fig. 1, *D* and *E*). Each hTCEpi cell line used throughout the study displays similar levels of c-Met receptor (Fig. 2*A*) using an antibody that was validated using positive and negative controls for c-Met expression (data not shown). We also observe full knockout of the CBL proteins in the -c-Cbl, -Cbl-b, and double KO (DKO) cell lines (Fig. 2*A*).

In Cas9 cells, c-Met phosphorylation is observed within 1 min of HGF treatment. Activity peaked around 30 min and tapered off over the next 12 h (Fig. 1, *A* and *C*). Total c-Met expression trends downward following HGF stimulation, indicative of ligand-mediated receptor degradation (Fig. 1, *A* and *B*). The expression of c-Met, its sensitivity to HGF, and its kinetics of phosphorylation and dephosphorylation are consistent with previous reports of c-Met signaling *in vitro* (55, 56). The parallel decreases in receptor phosphorylation and expression are consistent with the desensitization process.

To test the hypothesis that desensitization limits c-Met signaling, we used CRISPR–Cas9 technology to knockout either c-Cbl, Cbl-b, or both from hTCEpi cells. Multiple independent clones were isolated and amplified. Two were chosen for experimental purposes, and no differences between clonal lines were observed. All knockout clones were indistinguishable from the control (Cas9) cells in terms of morphology and basal growth rates. Knockout of c-Cbl and Cbl-b did not change the basal levels of c-Met expression (Fig. 2*A*), consistent with what has been observed with single c-Cbl knockout and the epidermal growth factor receptor (EGFR) (57). Knockout of the E3 ligases reduced the amount of detectable c-Met after Ub immunoprecipitation only in DKO cells, even with greater receptor activation (Fig. 2, *B* and *C*). Mouse immunoglobulin G immunoprecipitation control showed no detectable Ub or c-Met in the pull down (Fig. 2, *D*–*F*). Our data indicate a Ub-specific immunoprecipitation and is consistent with the study by Schwertman *et al.* (58).

**Figure 3:**
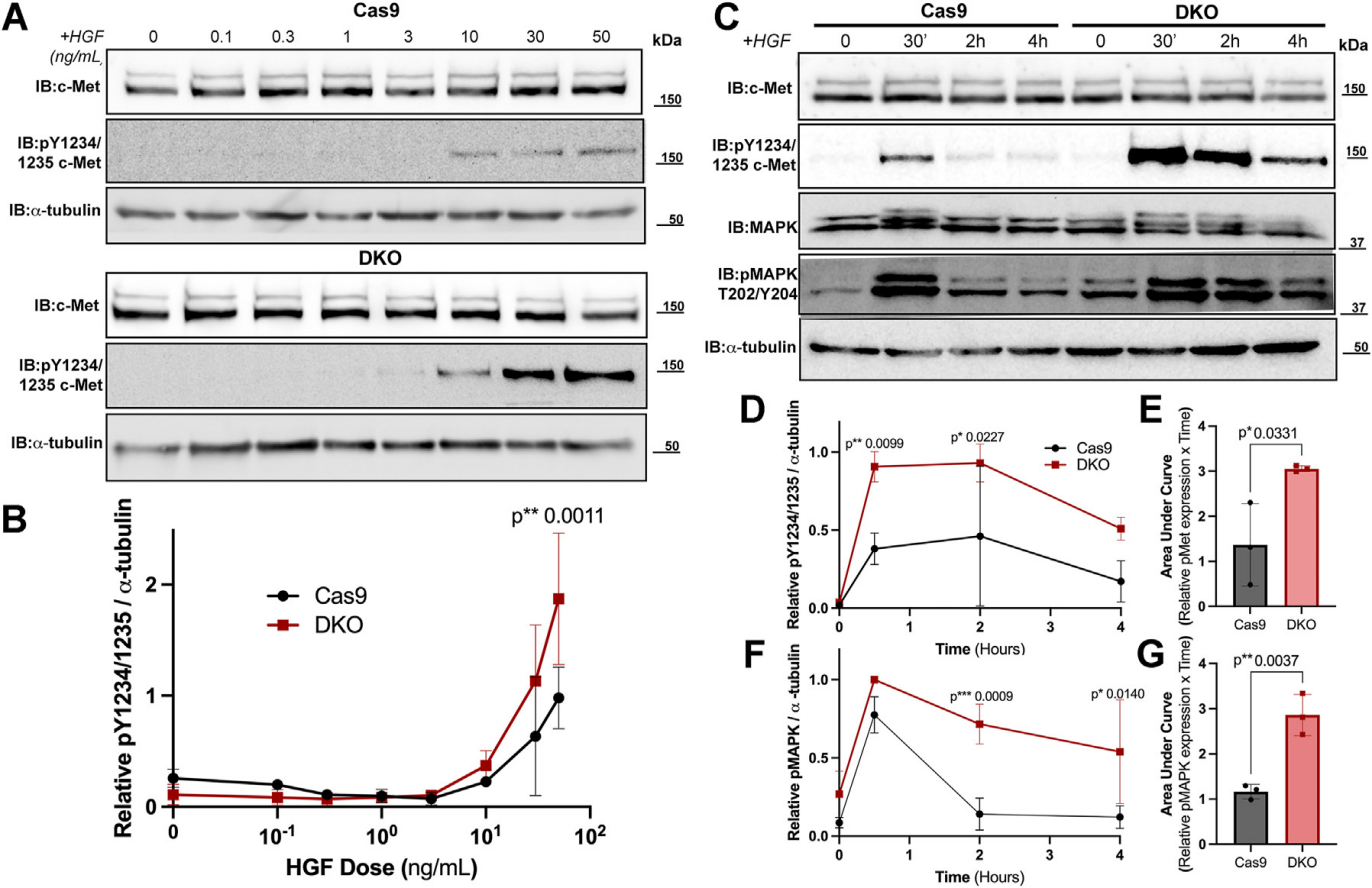
**HGF-mediated c-Met signaling is enhanced in magnitude and duration when both c-Cbl and Cbl-b are knocked out.***A*, cell lysates were prepared from Cas9 and DKO cells treated with increasing doses of HGF (0–50 ng/ml) for 30 min. Lysates (20 μg) were resolved by SDS-PAGE, transferred to nitrocellulose, and immunoblotted using antibodies against total c-Met, phosphorylated c-Met (pY1234/1235), and α-tubulin as a loading control. Shown are representative blots from experiments repeated three times. *B*, phosphorylated c-Met immunoreactive bands in (*A*) were quantified and normalized to α-tubulin. Graphed are average ± SD. Data were analyzed *via* a two-way ANOVA. *C*, Cas9 and DKO cells were treated with HGF (50 ng/ml) and harvested at the indicated time points (0–4 h). Lysates (20 μg) were resolved, and membranes were immunoblotted for total c-Met, phosphorylated c-Met (pY1234/1235), total p42/p44 MAPK (ERK1/2), phosphorylated p42/p44 MAPK (ERK1/2) (pT202/Y204), and α-tubulin. Shown are representative blots from experiments repeated three times. *D*, phosphorylated c-Met immunoreactive bands in (*C*) were quantified and normalized to α-tubulin. Graphed are average ± SD. Data were analyzed *via* a two-way ANOVA. *E*, area under the curve was calculated using Prism software for each individual time course. Graphed are average ± SD and individual data points (N = 3) for each cell line. Data were analyzed *via* an unpaired *t* test. *F*, phosphorylated MAPK blots were analyzed and graphed as described in (*D*). *G*, area under the curve for phosphorylated MAPK was calculated and graphed as in (*E*). DKO, double KO; HGF, hepatocyte growth factor; MAPK, mitogen-activated protein kinase.

All knockout cells had enhanced c-Met signaling; however, only in the DKO cells was this response statistically significant (Fig. 2, *G* and *H*). First, DKO cells were more sensitive to HGF treatment than Cas9 cells, as indicated by HGF dose–response curves (Fig. 3, *A* and *B*). Second, DKO cells exhibit increased activity and sustained c-Met signaling when subject to a 4 h time course of HGF treatment (Figs. 2, *G* and *H* and 3, *C*–*E*). Further investigation into a downstream effector of c-Met, p42/p44 mitogen-activated protein kinase (MAPK) (ERK1/2), also demonstrated sustained activity in the DKO cells (Fig 3, *A*, *F*, and *G*). Analysis of the time course curves indicated there is a 2.3-fold increase in average area under the curve (AUC) for phosphorylated c-Met in DKO cells compared with Cas9 cells (Fig. 3*E*).

**Figure 4:**
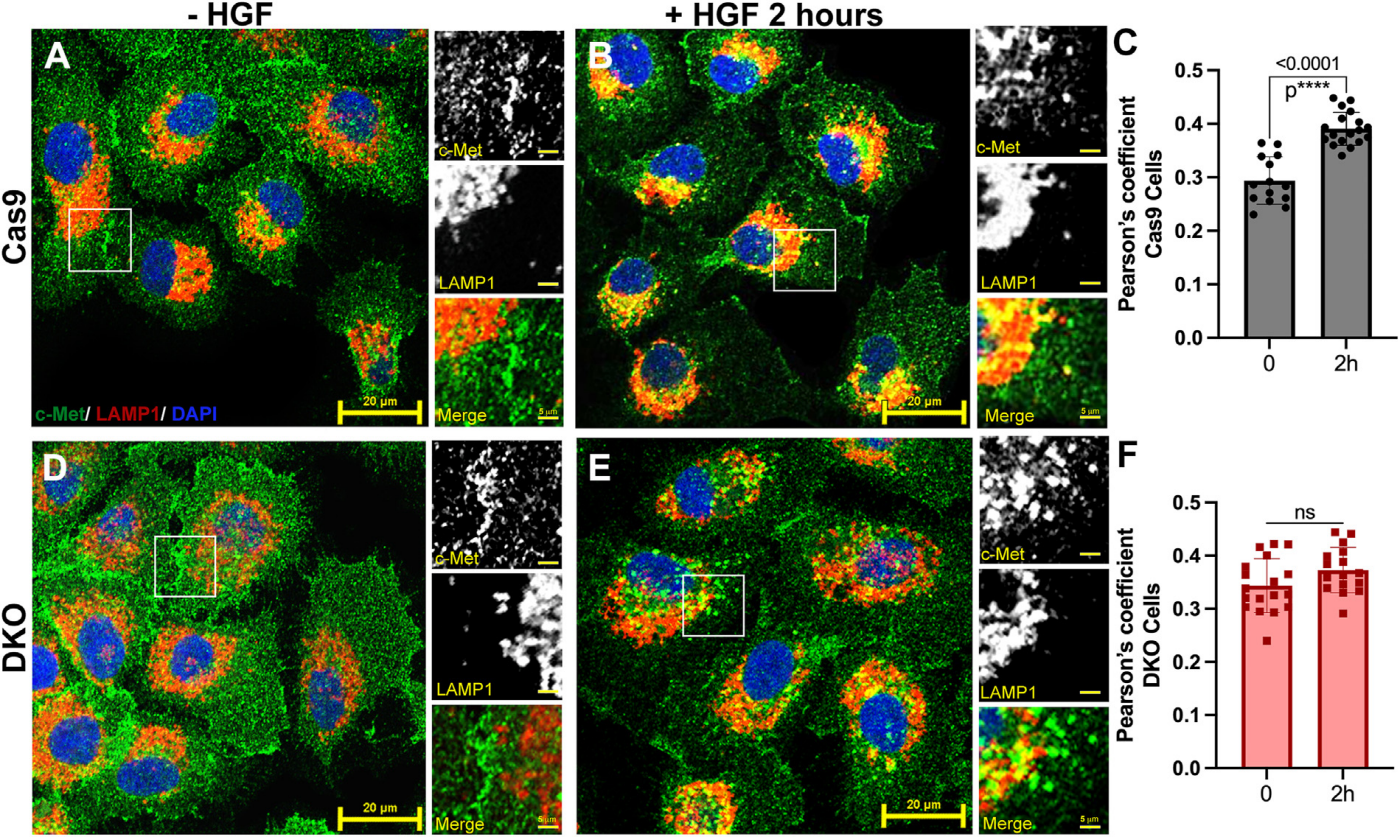
**c-Met trafficking is disrupted with loss of Cbl proteins.** Cas9 and DKO cells were seeded on 12 mm coverslips and grown for 48 h. Cells were treated with HGF (50 ng/ml) for the indicated time points (0–2 h) and processed for indirect immunofluorescence as described in the Experimental procedures section (c-Met primary: catalog no.: AF276, R&D Systems, secondary: Alexa-488, catalog no.: A-11055, Thermo; LAMP1 primary: catalog no.: 9091, Cell Signaling Tech, secondary: Alexa-568, catalog no.: A-10042, Thermo). Coverslips were mounted in ProLong Gold antifade reagent with DAPI and cured overnight before viewing at 60× objective on a confocal microscope. *A*, untreated Cas9 cells. Per each representative image, see *inset* magnified to the *right* with *green* (c-Met), *red* (LAMP1), and merged channels, where *yellow* indicates colocalization of the two proteins. *B*, Cas9 cells treated with HGF for 2 h. *C*, 400 cells total from three replicate experiments (90–200 per experiment) were analyzed per condition. Pearson’s coefficient was found for each image using the JaCOP plugin (82) in ImageJ. Graphed are average ± SD and individual data points. Groups were analyzed *via* unpaired *t* test. *D*, untreated DKO cells. *E*, DKO cells treated with HGF for 2 h. Scale bar for large images represent 20 μm; scale bar for *inset images* represent 5 μm. *F*, quantification and graphing of DKO cell Pearson’s coefficient as done in (*C*). DAPI, 4′,6-diamidino-2-phenylindole; DKO, double KO; HGF, hepatocyte growth factor; LAMP1, lysosomal-associated membrane protein 1.

For many RTKs (*e.g.*, EGFR, TrkA), c-Cbl has a role in their endocytic trafficking (57, 59, 60, 61). The significant increase in c-Met signaling coupled with the loss of detectable ubiquitylation drove us to ask if these changes were a consequence of altered trafficking. To assess this, we performed indirect immunofluorescence to monitor the subcellular localization of c-Met and lysosomes in Cas9 and DKO cells treated with HGF for 0 to 2 h. In Cas9 cells, HGF treatment causes a time-dependent redistribution of c-Met from the cytosol–plasma membrane to a more perinuclear location, overlapping with lysosomal-associated membrane protein 1 (LAMP1)–positive compartments. We observed a greater amount of colocalization in our Cas9 cells with HGF treatment (see *merge inset* of Fig. 4*B*). HGF-treated DKO cells had similar redistributions but with slower kinetics (Fig. 4). We observe a shift of c-Met from the cell surface to more perinuclear locations, but with less LAMP1 overlap, which is consistent with c-Met trafficking through the early endosome. We observe distinct c-Met foci and distinct LAMP1 foci rather than yellow staining, which indicates colocalization (see *merge inset* of Fig. 4*E*). Change in Pearson’s coefficient was not significant between the untreated and treated DKO conditions, whereas there was a statistically significant increase in colocalization between time points in our Cas9 cells (Fig. 4, *C* and *F*).

**Figure 5:**
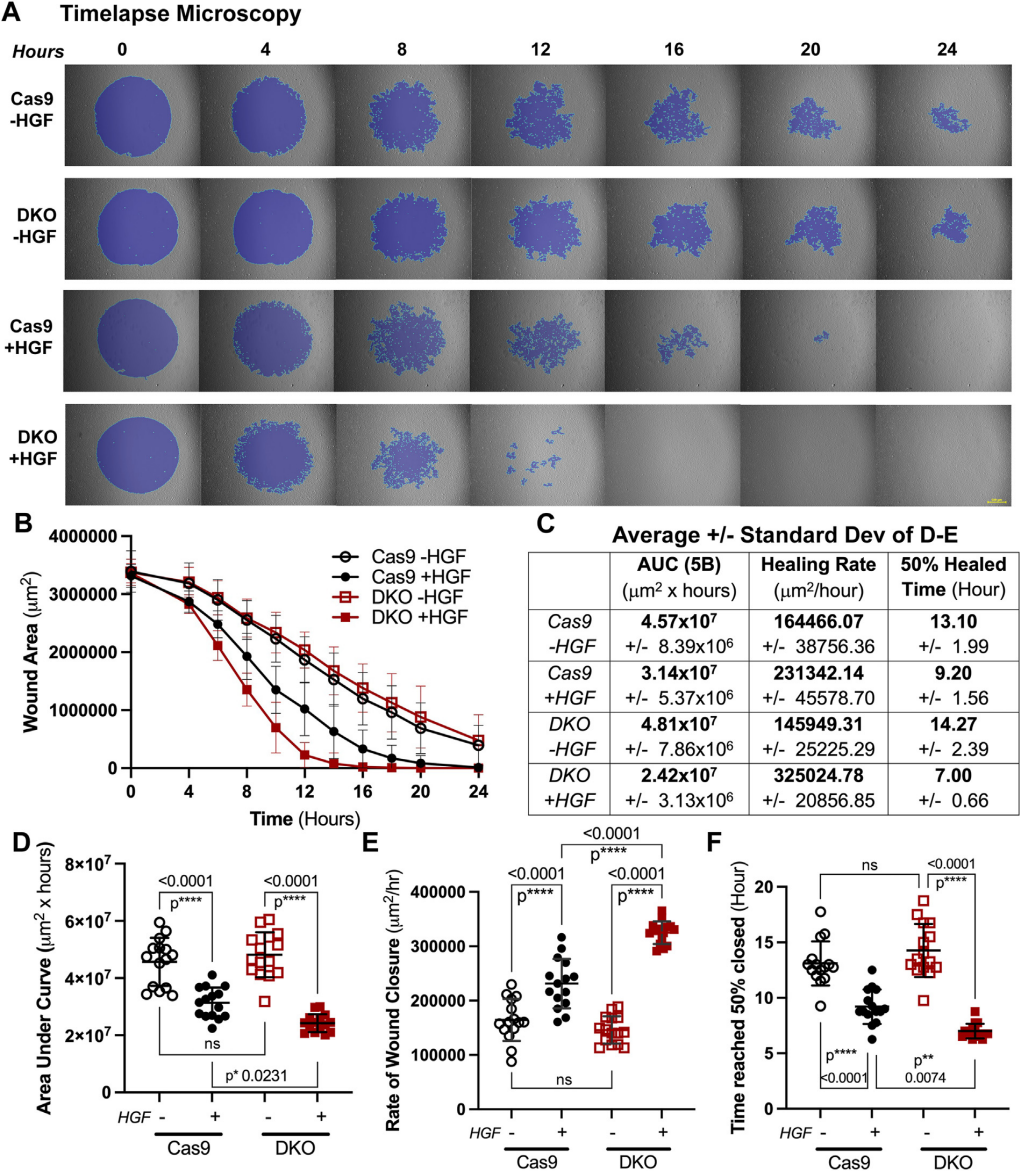
**HGF accelerates *in vitro* corneal epithelial wound healing, and loss of Cbl proteins potentiates the response.** Cas9 and DKO cells were seeded around 2 mm silicone plugs in a 6-well dish. Cells were grown to 95% confluency, and the plugs were removed, leaving acellular areas. Cells were treated with 50 ng/ml HGF (+HGF) or not (−HGF) and imaged in a Keyence BZ-X800 time-lapse microscope every 15 min for 24 h. Videos are included in Fig. S2. Wound area every 2 to 4 h was analyzed using the Keyence software (N = 15 for each condition). *A*, individual images from time-lapse video microscopy with the analyzed area shown in *purple*. Shown are representative images from 15 technical replicates from 5 to 6 experiments. Scale bar represents 500 μm. *B*, the average and SD of each analyzed area at each time point. *C*, table showing the averages and SDs for each of the graphs in (*D* and *E*). *D*, area under the curve was calculated using Prism software for each wound. Graphed are average ± SD and individual data points for each cell replicate. *E*, rate of absolute wound closure: the slope of the line between 4 and 12 h from (*B*). *F*, the time at which each wound reached 50% healed. Data in graphs (*D* and *E*) were analyzed *via* a two-way ANOVA. DKO, double KO; HGF, hepatocyte growth factor.

Seeing sustained c-Met signaling and slowed trafficking drove us to ask what cellular effects we would see with HGF treatment in DKO cells. To determine this, we examined the HGF-driven wound healing response with an *in vitro* wound healing assay (Fig. 5*A*). Cell movement into a 2 mm acellular area was monitored with time-lapse imaging in the presence of HGF or not. Images were collected every 15 min for 24 h (see Fig. S2 for videos). The remaining acellular area was quantified every 2 to 4 h (Fig. 5*A*). The wound areas (μm^2^) were graphed, and the AUCs were calculated (Fig. 5, *B*–*D*). Untreated Cas9 and DKO cells healed at an indistinguishable rate over the course of 24 h (Fig. 5, *B* and *C*). Treated DKO cells had the smallest AUC value compared with each condition (Fig. 5, *C* and *D*), indicating the fastest time to wound closure. When Cas9 cells were treated with 50 ng/ml HGF, their healing rate increased about 1.5-fold from untreated rate (Fig. 5, *C* and *E*). Knockout of c-Cbl and Cbl-b potentiate that effect: when subject to HGF treatment, DKO healing rate more than doubled their matched untreated rate (Fig. 5, *C* and *E*). Again, both untreated conditions reached 50% wound closure around 13 h (Fig. 5, *C* and *F*). Treated Cas9 cells reached 50% healed at 9.2 h, and treated DKO cells reached it the quickest at 7 h (Fig. 5, *C* and *F*).

## Discussion

The goal of this study was to identify potential pharmacological targets for accelerating corneal re-epithelialization. We used an established mediator of epithelial regeneration, the HGF–c-Met signaling axis, to determine if we could further enhance receptor activity by preventing the receptor desensitization that accompanies ligand stimulation. Specifically, we wanted to test the hypothesis that the E3 ligases c-Cbl and Cbl-b negatively regulate c-Met activity, and that knockout of the proteins will enhance c-Met signaling and ultimately corneal epithelial regeneration.

In hTCEpi c-Cbl and Cbl-b knockout (DKO) cells, there was a loss of detectable ligand-mediated c-Met after Ub immunoprecipitation (Fig. 2, *B* and *C*) and slowed ligand-mediated c-Met trafficking (Fig. 4). The effect of HGF treatment coupled with the loss of c-Cbl/Cbl-b ultimately resulted in enhanced c-Met receptor signaling as indicated by an increase in the magnitude and duration of receptor and effector (p42/p44 MAPK (ERK1/2)) phosphorylation (Fig. 3, *C*–*E*). Single knockout cell lines (hTCEpi -c-Cbl and hTCEpi -Cbl-b cells) show partially enhanced c-Met signaling (Fig. 2, *G* and *H*) and partially decreased ubiquitylation (Fig. 2, *B* and *C*) though neither measure was significantly different from control.

In our DKO cells, we observe a twofold increase in HGF-dependent *in vitro* wound healing rate (Fig. 5). Untreated Cas9 and DKO cells *in vitro* wound healing rates were indistinguishable from each other. Because we see no changes between untreated Cas9 and DKO healing rates or basal receptor activation, our results identify E3 ligases as highly attractive therapeutic targets because they only have effects in the presence of a separate activated (*i.e.*, *phosphorylated* c-Met) protein.

These findings build upon findings from other growth factor receptors (*e.g.*, EGFR (57, 62, 63), TrkA (61, 64, 65), IGFR1 (66, 67), and FGFR2b (68)) that are negatively regulated by E3 ligases. While much of this work has been done in cancer cell lines, there is emerging evidence that receptors in the corneal epithelium share the same mechanism of desensitization. For instance, work with the EGFR has demonstrated that inhibition of c-Cbl-mediated ubiquitylation accelerates ligand-driven corneal re-epithelialization and slows receptor internalization (57, 62). The effects we observed with c-Cbl and Cbl-b knockout have a more pronounced effect on c-Met receptor phosphorylation than what has been reported for the EGFR with c-Cbl knockout alone (57). Together, these data highlight the potential for c-Cbl/Cbl-b-specific antagonists as therapeutic agents for re-epithelialization. In addition, most ocular regenerative compounds can be administered topically, which allows for a high localized dose that would be significantly diluted if it entered the systemic circulation. This process alleviates concerns about enhanced growth factor activity because of defective ubiquitylation that is associated with many cancers (47, 69, 70, 71, 72).

The knockout of c-Cbl/Cbl-b showed a 90% loss of detectable c-Met after Ub immunoprecipitation, indicating that if other E3 ligases (*e.g.*, Cbl-3 and Nedd4 (57)) modulate c-Met signaling in the corneal epithelium, their contribution is minor. In the literature, c-Cbl is ascribed to be the primary E3 ligase for c-Met (73). However, our data indicate that c-Cbl and Cbl-b are functionally redundant, which has been reported for other RTKs (74, 75, 76). The amino acids of c-Cbl that bind c-Met (73) are 100% homologous in Cbl-b (73, 77). This is consistent with the notion that both E3 ligases can ubiquitylate c-Met. We cannot exclude the possibility that other E3 ligases ubiquitylate c-Met; however, the DKO of c-Cbl/Cbl-b was sufficient to lead to a physiologically significant increase in receptor signaling.

Although this study provides strong evidence that ubiquitylation negatively regulates c-Met signaling, where it acts specifically is unknown. While we cannot rule out the possibility that there is reduced phosphatase activity, we observed a slowed receptor redistribution to lysosomal compartments in DKO cells (Fig. 4) and believe this accounts for the enhanced receptor phosphorylation (Fig. 3, *C* and *D*). The reduction in c-Met colocalization with LAMP-1-positive compartments could be due to disrupted internalization, lysosomal degradation, or both (45, 46, 78, 79). While slowed endocytosis will decrease the rate of lysosomal degradation, work by Hammond *et al.* (80) has shown an increase in recycled c-Met when lysosomal degradation was prevented. Although our assays lack the sensitivity to quantitively assess the rates of receptor degradation, the enhanced c-Met phosphorylation provides compelling evidence that inhibition of c-Cbl/Cbl-b is a viable target to increase c-Met activity.

Many, if not all, RTKs are able to be ubiquitylated (73). Our data indicate that c-Met is monoubiquitylated in human corneal epithelial cells (Fig. 2*B*), consistent with work by Carter *et al.* (46). Jeffers *et al.* (77) reported a detectable immunoreactive band in stimulated c-Met receptor immunoprecipitations when using a poly-Ub-specific antibody (catalog no.: U5379; MilliporeSigma). However, polyubiquitylated c-Met was not observed in our cell lines. The composition of our Ub lysis buffer does not preclude the possibility that we have immunoprecipitated a protein complex comprised of another ubiquitylated protein that associates with c-Met. However, we believe c-Met is directly ubiquitylated and was pulled down properly based on the 150 kDa bands and previous reports of c-Met ubiquitylation (47, 49, 74).

Our *in vitro* model only examines the earliest steps in corneal re-epithelialization. More sophisticated models are needed to fully recapitulate the more complex aspects of corneal epithelial biology, including inflammation and fibrosis, corneal nerve health and regeneration, the fully differentiated epithelium, low-level HGF expression in tears, and the contributions of other growth factors. Omoto *et al.* (20) have shown *in vivo* that epithelial debridement murine models treated with HGF have enhanced re-epithelialization while also suppressing inflammation and opacification. We believe suppression of E3 ligase activity will further enhance these effects, thereby alleviating patient discomfort, risk of infection, and potential for blindness. Testing this hypothesis would require generating c-Cbl/Cbl-b antagonists and/or tissue-specific knockout mice, processes that are both labor and time intensive. Nonetheless, these questions must be answered to fully understand the effects of Cbl regulation on corneal biology and ocular tissues.

Our model provides a mechanistic examination of c-Met signaling in corneal epithelial cells, but *in vivo* work is needed to understand the role of c-Cbl/Cbl-b on HGF-driven cell migration on matrices, corneal nerve health and regeneration, and fibrosis. Our data quantify the role of c-Cbl/Cbl-b as negative regulators of c-Met signaling and how knockout of these proteins can potentiate receptor signaling. These studies lay the foundation for developing new Cbl-selective antagonists that have the therapeutic potential of promoting corneal epithelial homeostasis.

## Experimental procedures

### Cell culture

hTCEpi (52) cells were obtained from Evercyte. All cells were grown in keratinocyte basal medium 2 (catalog no.: CC-3103; Lonza) with growth supplements (hydrocortisone, transferrin, epinephrine, BPE, hEGF, and insulin; catalog no.: CC-4152; Lonza) and penicillin–streptomycin (catalog no.: 15140-122, ThermoFisher) at 37 °C with 5% CO_2_. Cells were propagated two times per week to maintain normal growth and never allowed to grow more than 90% confluent to prevent differentiation and quiescence of cells.

### Generation of knockout cells

hTCEpi cells were edited using CRISPR–CAS9 engineering technology to generate the c-Cbl/Cbl-b knockout cell lines. Briefly, cells were transduced with a lentivirus encoding CAS9 and the blasticidin-resistance gene. Blasticidin-resistant colonies were isolated, and a subset of these cells were transfected with tracrRNA and guideRNA against either c-Cbl, Cbl-b, or both (*tracrRNA:* mA∗mG∗CAUAGCAAGUUAAAAUAAGGCUAGUCCGUUAUCAACUUGAAAAAGUGGCACCGAGUCGGUGCU∗mU∗mU; *c-Cbl crRNA sequence 1*: mC∗mA∗UCUUUACCCGACUCUUUCGUUUUAGAGCUAUG∗mC∗mU; *c-Cbl crRNA sequence 2:* mC∗mU∗AUUCUUUAGCGCCAGCUUGUUUUAGAGCUAUG∗mC∗mU; *Cbl-b crRNA sequence 1:* U∗mG∗CACAGAACUAUCGUACCAGUUUUAGAGCUAUG∗mC∗mU; *Cbl-b crRNA sequence 2:* U∗mA∗AUCUGGUGGACCUCAUGAGUUUUAGAGCUAUG∗mC∗mU). Cells were seeded at a low density, and individual colonies were isolated, amplified, and screened *via* immunoblot to confirm the absence of proteins. Three positive clones of each cell line were identified. Single c-Cbl knockout cells are denoted as -c-Cbl cells. Single Cbl-b knockout cells are referred to as -Cbl-b cells. Cells with both c-Cbl and Cbl-b knockout are denoted as DKO cells. All control cells in this study are hTCEpi cells only expressing the Cas9 and blasticidin gene without guide or tracrRNA; these are referred to as Cas9 cells. Cas9 cells do not show any differences in growth morphology, growth rate, or receptor levels compared with the parental hTCEpi cells obtained from Evercyte (Figs. S1 and 2A). For experiments, two independent knockout clones were used to perform experiments.

### Serum starvation

Before all experiments requiring HGF treatment, cells were washed twice with PBS (pH 7.3) and incubated in keratinocyte serum-free media (KSFM) for at least 2 h.

### Immunoblots

Serum-starved cells were grown to confluence before treatment with human HGF (catalog no.: CYT-244; ProSpec). Ligand concentrations and treatment times are indicated in figure legends. Cells were subject to two washes of PBS in room temperature followed by equilibration to 4 °C for 5 min. Cells were harvested in radioimmunoprecipitation assay buffer (150 mM sodium chloride, 1% NP-40, 0.5% sodium deoxycholate, 0.1% SDS, 10 mM sodium pyrophosphate, 100 mM sodium fluoride, 50 nM Tris [pH 8.0]) supplemented with protease inhibitor PMSF (2 mM; Calbiochem) and solubilized by end-over-end rotation for 10 min at 4 °C. Insoluble material was removed *via* centrifugation for 10 min at 4 °C. The resulting cell lysate was assessed by bicinchoninic acid assay (catalog no.: 23225; Thermo Scientific), diluted in SDS sample buffer, and boiled for 3 min prior to gel loading. Equal amounts of protein were loaded and resolved by 10% SDS-PAGE before transfer to nitrocellulose membrane. Membranes were immunoblotted using the indicated antibodies and the manufacturer’s instructions: total c-Met (catalog no.: 8198), phosphorylated c-Met pY1234/1235 (catalog no.: 3077), c-Cbl (catalog no.: 2747), Cbl-b (catalog no.: 9498), total p42/p44 MAPK (ERK1/2) (catalog no.: 4695) phosphorylated p42/p44 MAPK (ERK1/2) (catalog no.: 9101) (Cell Signaling Technologies), and α-tubulin (catalog no.: 6199) (MilliporeSigma). Following incubation with the appropriate horseradish peroxidase–conjugated secondary antibody (antimouse: catalog no.: 31450; Invitrogen; anti-rabbit: catalog no.: 7074; Cell Signaling Technologies), enhanced chemiluminescence was used to visualize immunoreactive bands in a Fotodyne imaging system. When comparing c-Met phosphorylation, samples were run on the same gel if possible (time courses, Figs. 2*G* and 3C). Otherwise, immunoblots were run, processed, and developed together (dose response, Fig. 3*A*). Each experiment was performed at least three independent times. Densitometry analysis was executed using the ImageJ software (National Institutes of Health). GraphPad/Prism 9 (GraphPad Software, Inc) was used for generating graphs and performing statistical analysis (57, 62, 75). Area under the curves were generated by plotting each individual experiment and finding the replicate areas before graphing in Prism.

### Transwell migration assay

Cas9 cells were washed twice in PBS, trypsinized, and washed twice in KSFM before resuspension at 1 × 10^6^ cells/ml. Cells were seeded at 100,000 cells/8.0 μm pore transwell insert (Corning; catalog no.: 3422) in 100 μl KSFM. Transwell inserts were placed into wells containing KSFM with or without HGF (0, 5, or 50 ng/ml) and incubated for 16 h. Membranes were washed and fixed in methanol. Cells on the upper membrane were gently removed with a cotton swab, and the membranes were isolated and mounted using ProLong Gold antifade reagent with 4′,6-diamidino-2-phenylindole (DAPI) (catalog no.: P36931; Invitrogen). Ten fields of view per condition at 20× were randomly selected per membrane, and DAPI-stained nuclei were counted. Averages from treated conditions were normalized to untreated conditions and graphed in GraphPad.

### c-Met ubiquitylation assay

Confluent hTCEpi Cas9, -c-Cbl, -Cbl-b, and DKO cells were serum starved and pretreated with 0.1 nM MG-132 (catalog no.: 2194s; Cell Signaling Technologies) for 20 min to inhibit the proteosome before a 30 min treatment with 50 ng/ml HGF. Cells were harvested in Ub -lysis buffer (0.5% Triton X-100, Tris [pH 7.5], 150 mM NaCl, 1 mM EDTA, 1 mM sodium orthovanadate, 10 mM sodium fluoride) supplemented with 2 mM PMSF and 16 μM G5 Ubiquitin isopeptidase inhibitor I (catalog no.: sc-356181; Santa Cruz Biotechnology). Cell lysates (∼1.10 μg/μl protein) were incubated with an anti-Ub antibody that recognizes both monoubiquitylated and polyubiquitylated lysine residues (catalog no.: ST1200; MilliporeSigma) overnight at 4 °C before followed by a 2 h incubation with protein A/G Plus-Agarose beads (catalog no.: sc-2003; Santa Cruz Biotechnology). Beads were isolated, and immunoprecipitates were washed three times in Ub lysis buffer before dissociation from the beads *via* boiling in 6× SDS buffer. Immunoprecipitates were resolved by 10% SDS-PAGE and immunoblotted for total c-Met (catalog no.: 8198) (Cell Signaling Technologies). Portions of the whole cell lysates were run in parallel and immunoblotted for total and phosphorylated c-Met (catalog no.: 3077) (Cell Signaling Technologies) to verify activation of the receptor in the knockout cells (57, 62). Detectable c-Met following immunoprecipitated ubiquitylated proteins was normalized to whole cell lysate c-Met and graphed in Prism. An additional control experiment was run where mouse immunoglobulin G antibody (catalog no.: 5415; Cell Signaling Tech) was used to pull down any proteins that it bound with (Fig. 2, *D*–*F*).

### c-Met trafficking

Cas9 and DKO cells were grown for 48 h on 12 mm round glass coverslips. Cells were serum starved and treated with 50 ng/ml HGF (0–2 h), washed with PBS++++ (0.5 mM CaCl_2_, 0.5 mM MgCl_2_, 2% bovine serum albumin, 0.1 mM glucose, PBS [pH 7.3]), and fixed in 4% paraformaldehyde/PBS++ (0.5 mM CaCl_2_, 0.5 mM MgCl_2_, PBS [pH 7.3]) at room temperature for 5 min and then 4 °C for 15 min. Excess formaldehyde was washed away with PBS++. Cells were permeabilized and blocked for 20 min at room temperature in 0.1% saponin/5% fetal bovine serum/PBS++. Coverslips were incubated for 1 h at room temperature with primary antibodies against c-Met (catalog no.: AF276; R&D Systems) and LAMP1 (catalog no.: 909; Cell Signaling Technologies). After washing, coverslips were incubated at room temperature for 1 h in the dark with fluorophore-conjugated secondary antibodies (donkey antigoat Alexa-488, catalog no.: A11055; donkey anti-rabbit Alexa-568, catalog no.: A10042, ThermoFisher) in the blocking buffer. Coverslips were washed six times in PBS++ prior to a rinse in double-distilled water to remove excess salts. Coverslips were mounted onto slides using ProLong Gold antifade reagent with DAPI (catalog no.: P36931; Invitrogen). Slides were cured overnight in the dark and examined using a Zeiss 510 laser scanning confocal microscope at 60× objective at the same channel settings (57, 76, 81). Each experiment was repeated three times, and 5 to 6 images were acquired from each time point. About 90 to 200 cells per each experiment were analyzed per time point *via* ImageJ for a total of 375 to 500 cells per condition. The JaCOP (**J**ust **a**nother **CO**localization **P**lugin (82)) on ImageJ was used to find Pearson’s coefficient between the *green* (c-Met) and *red* (LAMP1) channels. Pearson’s coefficients were graphed in GraphPad and the 2 h time point compared with the untreated condition.

### *In vitro* wound healing assay

Silicone elastomer base (Sylgard 184 Elastomer; Dow Corning) was made per manufacturer’s instructions and cured for 96 h. Four 2 mm diameter silicone punch outs were placed directly on the bottom of a 6-well plate and spaced at least 2 mm apart. Cells were seeded around the plugs and incubated for 48 h with a media change at 24 h to achieve 95% confluency. The silicone plugs were removed following serum starvation, and the cells were rinsed with PBS to remove debris, creating a 2 mm uniform acellular area on the plate. Cells were kept in SFM or treated with 50 ng/ml of HGF and imaged using a BZ-X800 Keyence All-in-One fluorescent microscope at 4× objective. “Wounds” were visualized for 24 h by taking brightfield images every 15 min. The Keyence software was used to create videos of the wound healing over 24 h (Fig. S2). All wounds were analyzed by finding the area (μm^2^) every 2 to 4 h, graphing, and finding the AUC. Using the graphed data, the slope between hours 4 and 12 was calculated to find the rate of wound closure. The time it took to reach 50% confluency was found by analysis of images. GraphPad/Prism was used for statistical analysis and generating graphs (62, 83).

### Statistics

All statistics were performed *via* GraphPad/Prism 9. For all data, a two-way ANOVA with multiple comparisons or unpaired *t* tests were performed. All data are shown as the average ± standard deviation with individual data points where applicable. A *p* value less than 0.05 was considered statistically significant: α = 0.05; ∗*p* <0.05, ∗∗*p* <0.01, ∗∗∗*p* <0.001, and ∗∗∗∗*p* <0.0001. Significant *p* values are reported in figures.

## Data availability

All data will be uploaded to Open Science Framework upon publication.

## Supporting information

This article contains supporting information.

## Conflict of interest

The authors declare that they have no conflicts of interest with the contents of this article.
