# Supplementary material for: Knockout of c-Cbl/Cbl-b slows c-Met trafficking resulting in enhanced signaling in corneal epithelial cells
Source: J Biol Chem. 2023 Sep 9;299(10):105233. doi: 10.1016/j.jbc.2023.105233 (PMC10622846; doi:10.1016/j.jbc.2023.105233)
Supplement: Supporting Figure S1 [file mmc1.pdf]

Supporting Information

Knockout of c-Cbl/Cbl-b slows c-Met trafficking resulting in enhanced signaling in corneal epithelial cells.

Kate Tarvestad-Laise, BS, MS and Brian P. Ceresa, PhD

Included materials:
1. Supporting Figure 1: Proliferation rates and brightfield images of parental hTCEpi cells compared to hTCEpi cells expressing Cas9.

Figure S-1

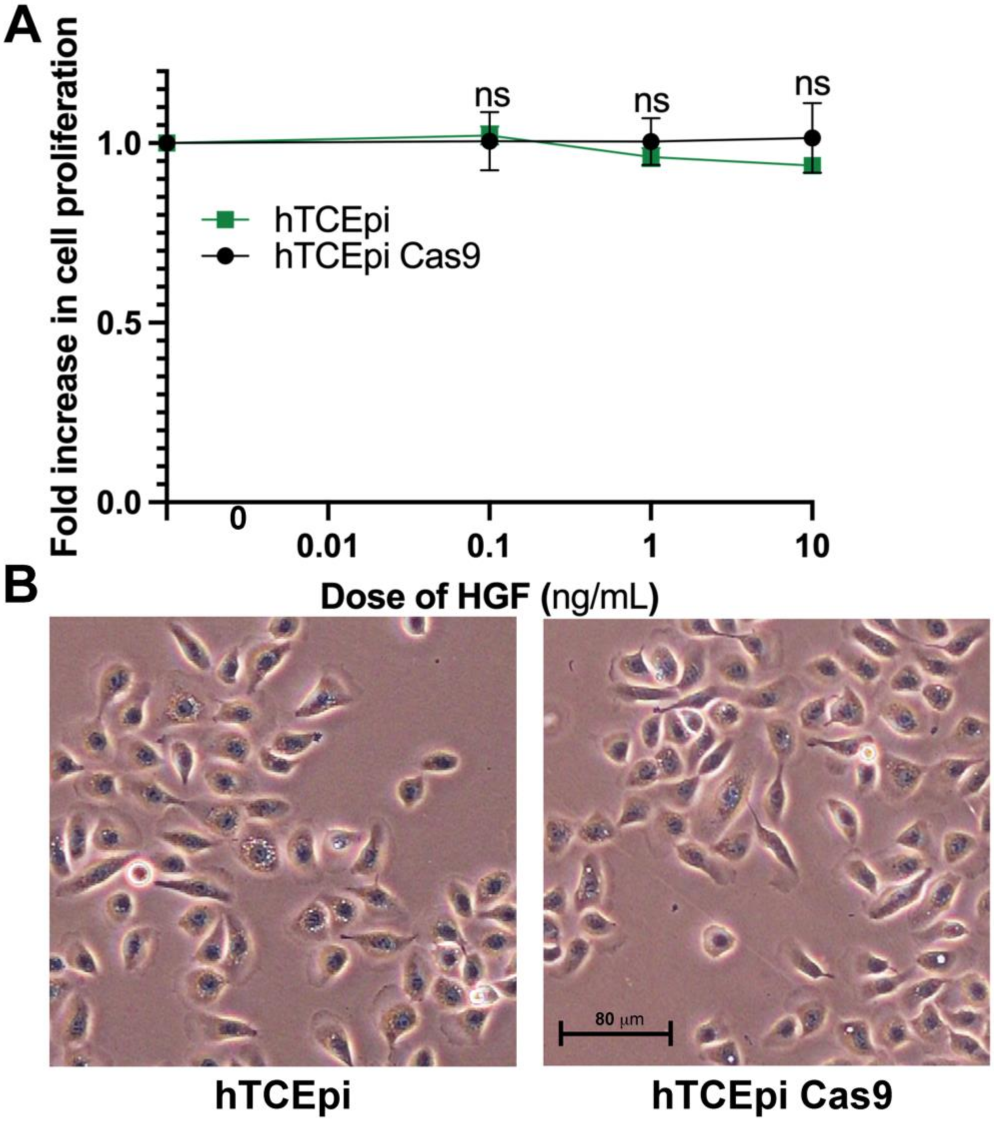

Supporting Figure 1. Transfection of hTERT- immortalized human corneal epithelial cells with Cas9 does not impact growth rate or cell morphology. A) hTCEpi-parental and hTCEpi-Cas9 cells were seeded in a 96-well plate and allowed to grow 24 hours. They were serum starved and treated with 0, 0.1, 1, or 10 ng/mL HGF for 24 hours before incubating with alamarBlue ® reagent (#BUF012A, Bio-Rad, CA) for 2 hours. The plate was read for cell-associated fluorescence on a Gen5 BioTek plate reader (excitation 530 nm; emission 590 nm). The experiment was preformed twice with 6 technical replicates per HGF dose. Data were normalized to untreated cells and plotted as the average +/- S.D. of proliferation. Data were subject to Unpaired t-tests. **B)** hTCEpi-parental and hTCEpi-Cas9 cells were seeded at 50k / plate and grown 24 hours. Brightfield images taken at 20X were obtained. Scale bar = 80 μm.
