# Supplementary material for: Knockout of c-Cbl/Cbl-b slows c-Met trafficking resulting in enhanced signaling in corneal epithelial cells
Source: J Biol Chem. 2023 Sep 9;299(10):105233. doi: 10.1016/j.jbc.2023.105233 (PMC10622846; doi:10.1016/j.jbc.2023.105233)
Supplement: Supporting Figure S2 [file mmc2.pdf]

Supporting Information

Knockout of c-Cbl/Cbl-b slows c-Met trafficking resulting in enhanced signaling in corneal epithelial cells.

Kate Tarvestad-Laise, BS, MS and Brian P. Ceresa, PhD

Included materials:

2. Supporting Figure 2: Videos of wound healing assay (Figure 5 includes still images). Videos are too large to attach in this file, so they are uploaded separately.

Files for Supplemental Figure 2 are uploaded as a .mp4 files individually.

For a preference of how to lay out the videos in the online format, see the still image below.

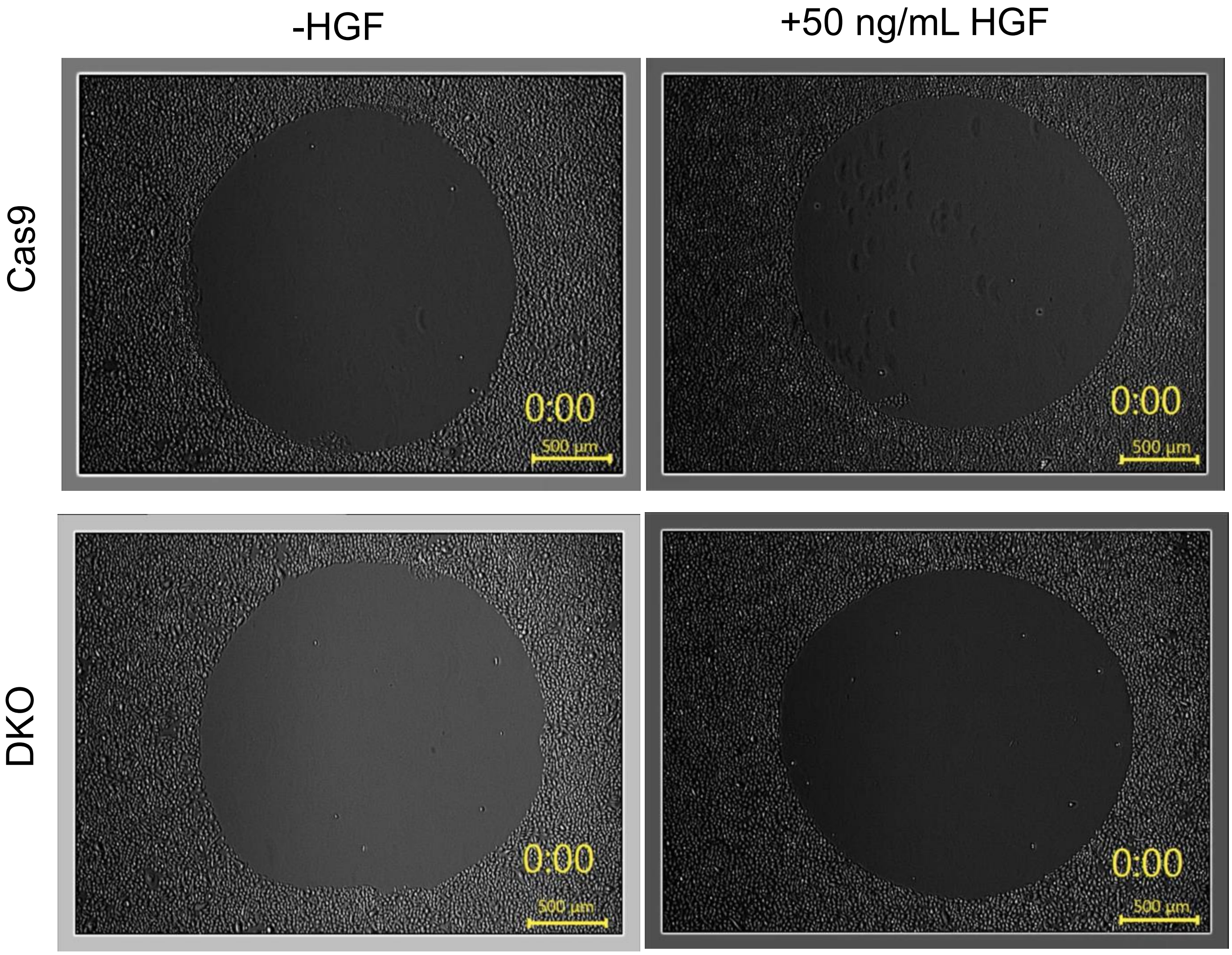

Supporting Figure 2. HGF accelerates *in vitro* corneal epithelial wound healing and loss of Cbl proteins potentiates the response. Videos of the still images seen in the main manuscript Figure 5. Scale bar = 500 μm.
